# An Update on Herbal Remedies for Treatment of Alveolar Osteitis: A Narrative Review

**DOI:** 10.1055/s-0045-1814462

**Published:** 2026-01-20

**Authors:** Sirinapa Siriwattanadom, Kantaporn Kheawfu, Kittipong Laosuwan, Phenphichar Wanachantararak, Warit Powcharoen

**Affiliations:** 1 Graduate School, Faculty of Dentistry, Chiang Mai University, Chiang Mai, Thailand; 2 Department of Pharmaceutical Sciences, Faculty of Pharmacy, Chiang Mai University, Chiang Mai, Thailand; 3 Research Center of Pharmaceutical Nanotechnology, Faculty of Pharmacy, Chiang Mai University, Chiang Mai, Thailand; 4 Department of Oral Biology and Diagnostic Sciences, Faculty of Dentistry, Chiang Mai University, Chiang Mai, Thailand; 5 Dental Research Center, Faculty of Dentistry, Chiang Mai University, Chiang Mai, Thailand; 6 Department of Oral and Maxillofacial Surgery, Faculty of Dentistry, Chiang Mai University, Chiang Mai, Thailand

**Keywords:** alveolar osteitis, dry socket, herbal products, medicinal plants

## Abstract

Alveolar osteitis (AO), commonly referred to as dry socket, is a frequent postoperative complication following tooth extractions. It is characterized by acute pain and delayed healing caused by disrupted blood clots and inflammation. Traditional treatments, such as irrigation and medicated dressings, have shown inconsistent success rates. Recently, herbal products have gained attention for their holistic approach in managing AO through their pharmacological properties. This study aimed to comprehensively overview recent clinical studies using herbal products in the treatment of AO. This study also discussed its phytochemical properties, and AO-related pharmacologic action of each herbal product. A literature review was conducted on the databases, consisting of PubMed, Scopus, Cochrane Library, and Google Scholar. The articles published from 2010 to 2024 were searched using key terms related to AO. Eight recent articles investigating the clinical efficacy of clove, turmeric, aloe vera, black cumin, and olive oil were selected according to eligible criteria. The findings revealed that these herbal products possess significant therapeutic properties, including anti-inflammatory and antibacterial effects, as well as wound-healing enhancement, contributing to improved clinical outcomes in AO management. Herbal products represent a promising alternative or adjunctive approach for the treatment of AO. Their therapeutic potential not only alleviates symptoms but also addresses underlying biological processes essential for tissue regeneration. Further research is recommended to establish standardized protocols and dose standardization, since most herbal products vary in bioactive concentration.

## Introduction

Alveolar osteitis (AO), or dry socket, is a common complication after tooth extraction, particularly third molars. It typically presents with severe pain 1 to 3 days post-extraction, often linked to clot disintegration in the socket. The most accepted etiology is Birn's fibrinolytic theory, where extraction trauma induces inflammation and tissue kinase release, leading to plasmin formation and subsequent clot dissolution. 1 Several studies suggest that bacteria may also contribute to AO development by promoting blood clot dissolution. 2

The management of AO varies among clinicians based on their expertise and experience in handling the condition. AO management primarily focuses on pain relief until healing occurs, with local treatment usually sufficient. Traditionally, AO is managed with irrigation and medicated dressings, such as Alvogyl, zinc oxide eugenol (ZOE), or antibiotic dressings, to provide pain relief and support healing.

In recent years, there has been growing interest in herbal therapies as alternative or complementary treatments for various medical conditions. 3 With advances in herbal medicine, reviewing recent developments in herbal products for managing AO is essential. Recent research provides insights into their efficacy, mechanisms, and safety. This article reviews literature from the past 15 years on herbal compounds for AO, focusing on their phytochemistry, mechanisms of action, wound-healing activity, and clinical performance.

## Methods

This narrative review followed the research question: “What does recent literature reveal about the clinical efficacy of herbal products in treating AO?” Inclusion criteria comprised peer-reviewed English studies on human subjects using herbal products with or without comparators, while exclusions were case reports, series, reviews, letters, conference abstracts, synthetic compounds, and non-plant derivatives. An electronic search of PubMed, Scopus, Cochrane Library, and Google Scholar (2010–2024) was conducted using Boolean operators ( Table 1 ). Two authors independently screened titles, abstracts, and full texts, with disagreements resolved by discussion. Data were extracted into a spreadsheet, including author, year, sample, herbal product, clinical measures, and findings. Phytochemistry, pharmacologic actions, efficacy, and adverse events were also reviewed from included studies and relevant references.

**Table 1:** The summary of search strategies among the databases

Database	Search strategies
PubMed	(“alveolar osteitis”[All Fields] OR “dry socket”[All Fields]) AND ((english[Filter]) AND (2010:2024[pdat]))
Scopus	(TITLE-ABS-KEY (dry AND socket) OR TITLE-ABS-KEY (alveolar AND osteitis)) AND PUBYEAR > 2009 AND PUBYEAR < 2025 AND (LIMIT-TO (LANGUAGE, “English”)) AND (LIMIT-TO (SRCTYPE, “j”)) AND (LIMIT-TO (DOCTYPE, “ar”))
Cochrane Library	#1 dry socket #2 Alveolar osteitis #3 #1 OR #2 with Cochrane Library publication date Between Jan 2010 and Dec 2024, in Trials
Google Scholar	allintitle: “Alveolar osteitis” OR “dry socket”

## Results and Discussion

**Fig. 1:**
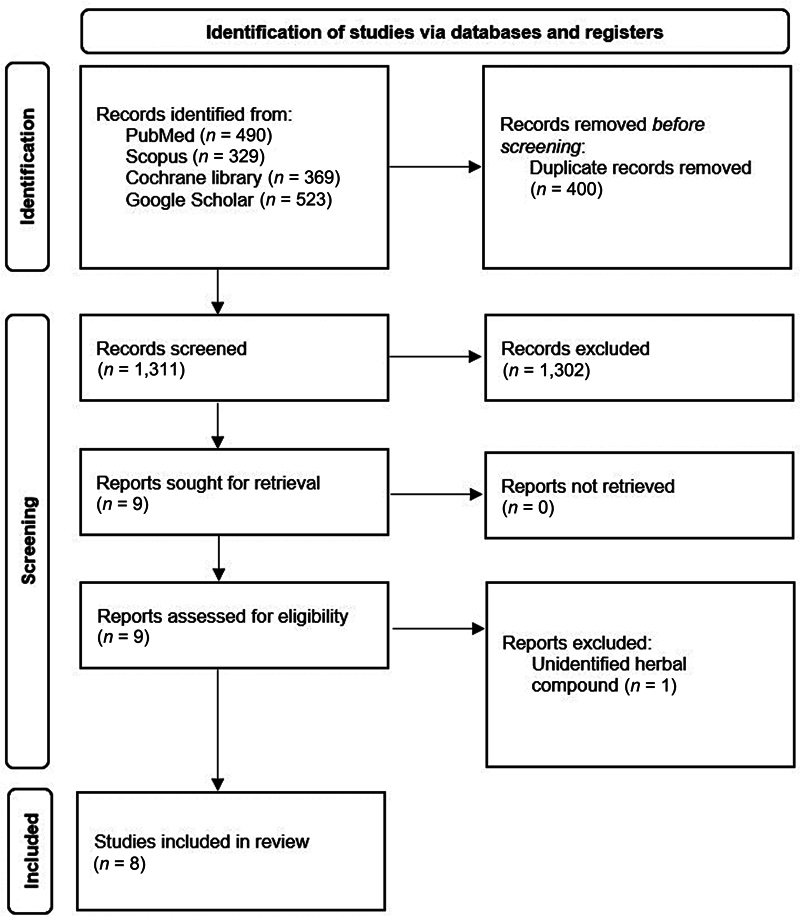
Flowchart showing the identification of the included studies.

The initial search identified a total of 1,711 articles. After elimination of duplicate articles, 1,311 articles remained. Nine articles were included after screening of titles and underwent full-text reading. Finally, eight articles were selected for this narrative review, of which one article was excluded because the herbal mixture could be identified ( Fig. 1 ). The summary results and discussions present an updated overview of herbal products including clove, turmeric, aloe vera, black cumin, and olive oil. A descriptive summary of the findings is presented in Table 2 .

**Table 2:** Summary of clinical studies on herbal compounds for the management of alveolar osteitis

Author (s) (year)	Participant's characteristic	Herbal products and comparators	Clinical measure(s)	Study finding(s)
Memon et al (2023) 38	50 patients (20 females, 30 males, 26–52 years)	Turmeric and mustard oil dressing versus Alvogyl dressing	Pre- and postoperative VAS and healing index (tissue color, palpation response, granulation tissue presence, and enhanced incision margins) on days 1, 2, and 3	Both groups experienced pain relief, with turmeric demonstrating accelerated wound healing compared with Alvogyl.
Khan et al (2023) 68	90 patients (68 females, 32 males, 18–52 years)	Intra-alveolar dressing of olive oil versus Nano-Bio Fusion (NBF) gel	Pain score, gingival erythema, granulation tissue, and the socket margin on baseline, and days 3, 5 and 7	NBF gel showed significantly lower VAS scores than olive oil at all time points. By day 7, NBF gel achieved healthy gingiva, while olive oil showed mild to normal healing. Both groups had significant margin healing, with absence of granulation tissue in 42 out of 45 NBF cases and 39 out of 45 olive oil cases.
Alabdullah et al (2023) 15	36 patients, 40 sockets (17 female, 19 males, 20–50 years)	Eugenol on a gel foam carrier versus *Nigella sativa* oil	Assessment of soft tissue healing and severity of inflammation at the first visit, and on days 3 and 7 after dressing.	Eugenol and *Nigella sativa* oil both enhanced wound healing and reduced inflammation, with *N. sativa* oil showing superior effectiveness, particularly on day 7.
Khan et al (2022) 53	60 patients (31 females, 29 males, 18–70 years)	Mixture of *Nigella sativa* 's powder and oil versus Alvogyl versus normal saline rinse	VAS was recorded at 5, 30, and 60 minutes after dressing, and patients were requested to note their pain scores for 7 days.	A mixture of *Nigella sativa* powder and oil showed a statistically significant improvement in relieving pain compared with the Alvogyl group.
Al-Niaimi et al (2022) 54	52 patients (25 females, 27 males, age not specified)	Admix of Miswak powder and *Nigella sativa* oil versus Alvogyl	The pain score was recorded in VAS on days 1, 2, and 3 after treatment.	The pain levels significantly decreased on days 2 and 3 in both groups. There were no notable differences in the pain scores between the two groups.
Ali et al (2021) 26	40 patients (25 females, 15 males, 29–60 years)	*Aloe vera* extract versus Alvogyl	Record the pain VAS score on days 2 and 7 after starting dressing.	*Aloe vera* extract is superior to Alvogyl in its capacity to decrease pain level.
Rajashri et al (2020) 39	40 patients (gender and age not specified)	*Curcuma longa* and pineapple extract versus zinc oxide eugenol	Record incidence and intensity of dry socket in each group and evaluate by scores	There was significant reduction in pain, inflammation, and discomfort after turmeric pineapple extract dressing and ZOE dressing. Wound healing was seen faster in the study group than the control group.
Lone et al (2018) 37	178 patients (gender and age not specified)	Turmeric dressing with mustard oil versus ZOE pastes	Record the number of patients with symptom improvement each day after receiving the dressing.	In the turmeric dressing group, all symptoms, including pain, swelling, and necrotic socket, began to subside significantly faster than in the ZOE group.

Abbreviations: VAS, visual analog scale; ZOE, zinc oxide eugenol.

### Clove

Clove ( *Syzygium aromaticum* L.) is widely used in dentistry for its analgesic, anti-inflammatory, and antimicrobial properties. Clove oil, a key component of ZOE and Alvogyl, is extensively utilized as a medicated dressing in the management of AO. Clove is rich in a variety of bioactive compounds, with eugenol being the most prominent constituent.

#### Antibacterial Properties of Clove

Eugenol exhibits broad-spectrum antibacterial activity, targeting a wide range of bacteria that may colonize the exposed socket and contribute to infection. Antimicrobial activity of eugenol due to the presence of a free hydroxyl group in the molecule. These functional groups can interact with the cytoplasmic membrane of microbial cells. Its disruption of the cell wall and membrane causes leakage of intracytoplasmic components. 4 Another mechanism involves the inhibition of ATPase, which induces mitochondrial dysfunction. This dysfunction leads to the production of intracellular reactive oxygen species (ROS) and metabolic disturbances, causing DNA damage and resulting in cell death. 5 Clove oil has been shown to inhibit oral pathogens effectively. 6

#### Anti-inflammation and Analgesic Properties

Clove oil regulates the immune response and reduces inflammatory symptoms by enhancing humoral immunity and reducing the release of inflammatory lymphokines. 7 Clove oil can inhibit inflammatory processes through the nuclear factor-kappa B (NF-κB) pathway, a key transcription factor involved in regulating the production of proinflammatory cytokines such as tumor necrosis factor α (TNF-α), interleukin-1 β (IL-1β), and interleukin 6 (IL-6). 8 Eugenol has been found to inhibit voltage-gated sodium channels (VGSC) in the primary supply neurons of the teeth. The analgesic and local anesthetic effects of eugenol can be modulated by its inhibitory effect on voltage-gated channels (Na ^+^ and Ca ^2+^ ) and activation of transient receptor potential vanilloid 1 (TRPV1). 9

#### Wound-healing Properties

Clove oil plays a significant role in stimulating wound healing by increasing fibroblast migration, accelerating wound closure, and promoting angiogenesis. Clove oil emulsion significantly enhanced wound healing in Wistar rats by promoting re-epithelialization, collagen formation, and dermal repair, as confirmed by histological analysis. 10 A eugenol hydrogel was shown to accelerate diabetic wound healing in mice by inhibiting the lectin-like oxidized low-density lipoprotein receptor-1 (LOX-1) and NF-κB pathways, upregulating vascular endothelial growth factor (VEGF), and downregulating matrix metalloproteinases 9 (MMP9) to promote angiogenesis and wound closure. 11

#### Clinical Efficacy of Clove in the Treatment and Prevention of AO

From the past, eugenol-soaked gauze has been used for managing severe pain in AO. 12 Previous systematic review found that eugenol was more effective than curettage and irrigation, and comparable to thermosetting gel and Salicept in reducing pain and inflammation. 13 A eugenol-based paste completely prevented AO and was also associated with decreased pain, inflammation, and infection, as well as enhanced wound healing. 14

However, among the included studies, clove appeared primarily as a comparator rather than the main intervention. A recent study found that while eugenol effectively reduced inflammation and promoted healing, *Nigella sativa* oil produced greater improvement by day 7. 15 This suggests that although clove remains clinically effective, its therapeutic advantage may not be superior when compared with certain plant-derived oils with broader anti-inflammatory mechanisms. Therefore, in the context of recent evidence-focused AO care, clove may serve as a reliable baseline treatment but not necessarily the most potent herbal option among those reviewed.

#### Toxicity and Adverse Effects of Clove

The United States Food and Drug Administration (FDA) categorizes clove oil as generally recognized as safe (GRAS), making it suitable for use in cosmetics, pharmaceuticals, and food products. 16 However, it may present some toxicity depending on the type of histological structure exposed to this compound and the concentration used. Some studies show cytotoxicity against human dental pulp fibroblasts and human fibroblasts. With topical use, some reports indicate that directly applying 100% clove oil can cause oral mucosa burns. 17

### *Aloe Vera*

*Aloe vera* ( *Aloe barbadensis* Miller), a member of the Asphodelaceae family, has long been applied in traditional medicine and is now recognized in oral health for its regenerative potential. *Aloe vera* contains a yellow latex rich in anthraquinones (aloin, aloe-emodin, chrysophanol) and an inner gel abundant in polysaccharides such as acemannan and glucomannans. 18

#### Antibacterial Properties of *Aloe Vera*

*Aloe vera* exhibits antibacterial activity against both gram-positive and gram-negative bacteria, primarily due to its anthraquinone and saponin content. Anthraquinones have a structural analog of tetracycline and they inhibit bacterial protein synthesis. Polysaccharides in *Aloe vera* gel have been attributed to direct antibacterial effects by activating phagocytic leukocytes to destroy bacteria. Additionally, *Aloe vera* contains pyrocatechol a hydroxylated phenol, known to have a toxic effect on microorganisms. 19 *Aloe vera* effectively inhibits the growth of oral pathogens such as *E. faecalis* , *P. intermedia* , *S. mutans* , and *P. anaerobicus* . 20 Since bacteria from the *Prevotella* and *Peptostreptococcus* groups are frequently associated with AO, *Aloe vera* demonstrates potential for reducing microbial involvement in this condition. 2

#### Anti-inflammation Properties of *Aloe Vera*

The anti-inflammatory properties of the *Aloe vera* are associated with the suppression of proinflammatory cytokines, including IL-8, IL-6, IL-1, and TNF, as well as the reduction of leukocyte adhesion. Emodin, emodinolin, and anthraquinone function as competitive inhibitors of thromboxane synthetase, prostaglandin E _2_ (PGE _2_ ) synthesis, and cyclooxygenase (COX) through the arachidonic acid pathway. Study has shown that aloin and aloe-emodin dose-dependently inhibited inducible nitric oxide synthase (iNOS) mRNA expression and nitric oxide (NO) production, while also suppressing COX-2 mRNA and PGE _2_ levels in lipopolysaccharide **(** LPS)-stimulated RAW 264.7 macrophages. 21

Bradykinin induces painful inflammation through vasodilation, while *Aloe vera* mitigates this response via 1-carboxypeptidase, which inactivates bradykinin and produces an analgesic effect. Additionally, *Aloe vera* reduces inflammation associated with prostaglandin synthesis and leukocyte infiltration. 22

#### Wound-healing Properties of *Aloe Vera*

Different mechanisms have been proposed for the wound-healing effects of *Aloe vera* gel, which include keeping the wound moist, increasing epithelial cell proliferation and migration, rapid maturation of collagen, increasing angiogenesis, and reduction in proinflammatory cytokine. 18

Polysaccharides from *Aloe vera* , such as glucomannan and acemannan, stimulate fibroblast and keratinocyte activity, enhancing collagen deposition and cytokine production. 23 In animal models, *Aloe vera* gel demonstrated strong angiogenic and wound-healing effects by increasing transforming growth factor-β (TGF-β), VEGF, basic fibroblast growth factor (bFGF), and peroxiredoxin 6 (Prdx6) expression, thereby promoting cell proliferation, tissue regeneration, and vascularization. 24 Consistently, *Aloe vera* hydrogel accelerated post-extraction healing in rats by suppressing macrophage activity during inflammation and enhancing fibroblast proliferation and growth factor expression. 25

#### Clinical Efficacy of *Aloe Vera* in the Treatment and Prevention of AO

One clinical trial among the included studies investigated *Aloe vera* for AO management. Hatem Ali et al demonstrated that *Aloe vera* gel or extract reduced pain scores more effectively than Alvogyl, with healing progressing more rapidly and without adverse effects. 26 Similarly, previous studies reported that *Aloe vera* extract significantly reduce pain in AO management compared with Alvogyl, and also showed significantly better healing. 27 Additionally, the development of acemannan hydrogel (SaliCept Patch) reflects clinical translation of these wound-healing properties and has been successfully applied to reduce the incidence of AO following tooth extraction. 28

These findings support *Aloe vera* 's clinically observable wound-healing behavior, such as enhanced epithelialization and fibroblast activity, which align with its polysaccharide-mediated stimulation of tissue repair. Importantly, the clinical outcomes correspond well with its known molecular properties, confirming that *Aloe vera* 's anti-inflammatory and wound-healing pathways translate meaningfully to patient recovery.

#### Toxicity and Adverse Effects of *Aloe Vera*

Clinical trials have not reported any considerable serious adverse effects from the use of *Aloe vera* . 29 Topical use of *Aloe vera* whole-leaf extract in humans can cause adverse clinical effects like hypersensitivity and skin allergic responses. These reactions are mostly due to anthraquinones, like aloin and barbaloin, 18 and adverse effects of oral supplements like cramping and diarrhea to those who are allergic to plants in the Liliaceae family. 29

### Turmeric

Turmeric ( *Curcuma longa* ), a member of the Zingiberaceae family, has long been used in dentistry. Its rhizomes contain volatile oils (turmerone, atlantone, zingiberene), sugars, proteins, resins, and polyphenolic compounds known as curcuminoids comprising curcumin, demethoxycurcumin, and bisdemethoxycurcumin with curcumin as the most active compound. Curcumin, also known as diferuloylmethane, exhibits keto-enol tautomerism that underlies its physicochemical stability and strong antioxidant activity. 30

#### Antibacterial Properties of Turmeric

Curcumin can also inhibit bacterial growth by targeting the bacterial cell membrane, cell wall, protein, and DNA. Curcumin can affect cytoplasmic membrane permeability and elicit destruction of the integrity of bacterial cell walls and disruption of the selectivity of osmosis in the bacterial cell membrane, eventually killing the bacteria. 31 Curcumin disrupts quorum sensing, thereby inhibiting biofilm formation, reducing biomass, and altering biofilm structure. 32

Curcumin has been shown to reduce the virulence of *P. gingivalis* by downregulating genes encoding key adhesins ( *fimA, hagA, hagB* ) and proteinases ( *rgpA, rgpB, kgp* ). It also exhibits inhibitory effects against other periodontal pathogens, including *A. actinomycetemcomitans, F. nucleatum, P. intermedia,* and *T. denticola,* the latter being closely associated with AO. 33

#### Anti-inflammatory Properties of Turmeric

Curcumin exerts anti-inflammatory effects by inhibiting key transcription factors, NF-κB and activator protein 1 (AP-1), thereby suppressing the production of proinflammatory cytokines, including TNF-α, IL-1β, IL-2, IL-6, IL-8, and PGE _2_ . It also downregulates enzymes such as 5-LOX, COX-2, and COX-5 involved in prostaglandin synthesis, and reduces nitric oxide production by inhibiting NOS enzyme expression. 34 Additionally, curcumin possesses properties that help alleviate pain. It selectively inhibits lipoxygenase, phospholipase A2, and COX-2, but not COX-1, thereby providing anti-inflammatory and analgesic benefits without the side effects associated with non-selective analgesics. Furthermore, it depletes substance P in nerve terminals, contributing to its analgesic effect. 34

#### Wound-healing Properties of Turmeric

Turmeric enhances wound healing through several mechanisms. One of the important mechanisms is stimulating blood vessel growth. Curcumin induces the expression of pro-angiogenic factors, including VEGF, TGF-β, and FGF-2. In an in vitro gingival fibroblast model, curcumin significantly upregulated TGF-β1 and VEGF. 35

Fibroblast migration is essential for granulation tissue formation and collagen deposition. Curcumin has been shown to stimulate fibroblast infiltration, proliferation, and collagen synthesis. A curcumin-loaded nanofibrous matrix further enhanced wound healing in rodents by accelerating fibroblast growth and increasing FGF expression. 36

#### Clinical Efficacy of Turmeric in the Treatment and Prevention of AO

Three included studies assessed turmeric as a dressing material. Lone et al and Memon et al both demonstrated that turmeric dressing (often mixed with mustard oil) provided faster symptom resolution than ZOE or Alvogyl. 37 38 Improvement in pain and granulation tissue formation was observed as early as day 2 in turmeric-treated sites. Rajashri and MP also reported that a turmeric–pineapple extract formulation significantly improved healing outcomes compared with ZOE. 39 These clinical findings align with curcumin's known mechanisms such as NF-κB pathway inhibition and enhancement of fibroblast proliferation, supporting turmeric's value both as an analgesic and wound-healing agent.

#### Toxicity and Adverse Effects of Turmeric

Turmeric is currently listed by the FDA as GRAS for use as a coloring and flavoring agent in food. Its widespread use in food even in high doses without known adverse effects suggests its safety. 40 Curcumin is well-tolerated and considered generally safe for dental use, although it can sometimes cause skin reactions such as contact dermatitis. 41 There have been some reports of tongue discoloration with the use of curcumin mouthwash due to the high concentration of yellow pigment, which easily adheres to the tongue surface. However, it was temporary staining and disappeared after brushing. 42

### Black Cumin

Black cumin ( *Nigella sativa* ) of the Ranunculaceae family has long been used for culinary and medicinal purposes due to its anti-inflammatory and immunomodulatory properties. Its essential oil contains thymoquinone, thymohydroquinone, thymol, carvacrol, and other bioactive compounds responsible for its therapeutic effects. Thymoquinone, the major constituent, exhibits potent anti-inflammatory and antimicrobial activities. 43 In addition, black cumin provides proteins, alkaloids, phenolics, and vitamins A, C, and E, along with B vitamins, which act as natural antioxidants and protect against lipid peroxidation. 44

#### Antibacterial Properties of Black Cumin

Thymoquinone, the principal active constituent, exerts antibacterial effects by generating ROS to target bacterial cells and disrupting membrane integrity, partly through efflux pump inhibition. 45 Thymoquinone shows strong antimicrobial activity against *P. gingivalis* , *A. actinomycetemcomitans* , and *P. intermedia* , reducing subgingival bacterial counts. 46 It also suppressed H _2_ S production, which is associated with bacterial membrane disruption, and downregulated major virulence factors in *F. nucleatum* and *P. gingivalis* . 43 Thymoquinone exhibits selective antimicrobial activity and enhances the efficacy of antibiotics such as tetracycline and benzalkonium chloride against resistant strains. By inhibiting bacterial efflux pumps, it increases intracellular antibiotic accumulation, thereby improving antibiotic effectiveness. 47

#### Anti-inflammatory Properties of Black Cumin

Black cumin and thymoquinone have also been experimentally shown to have noticeable anti-inflammatory functions. Houghton et al demonstrated that black cumin oil significantly inhibited the COX and 5-LOX pathways in arachidonate metabolism within rat peritoneal leukocytes, resulting in reduced formation of thromboxane B2 (TXB2) and leukotriene B4 (LTB4) metabolites. 48 Thymoquinone was shown to block LPS-induced activation of p38 mitogen-activated protein kinase (p38-MAPK), ERK1/2, and NF-κB, leading to suppressed expression of proinflammatory mediators IL-1β, TNF-α, MMP-13, COX-2, and PGE _2._ 49 Thus, thymoquinone likely exerts its anti-inflammatory effects by modulating several key factors involved in multiple signaling pathways, including the NF-κB pathway, p38 MAPK pathway, and ERK1/2 pathway. 50

#### Wound-healing Properties of Black Cumin

Black cumin enhances cell viability in dermal fibroblasts (NHDF) and endothelial cells (HUVECs) by upregulating VEGF and PDGF, thereby promoting angiogenesis and cell proliferation. It also stimulates fibroblast activity and collagen synthesis, which are essential for tissue strength and wound repair. 51 In addition to enhancing soft tissue healing, black cumin also promotes new bone formation and accelerates bone regeneration. A previous study on rabbits found that 2 weeks after tooth extraction, treatment with black cumin oil led to more active bone formation in the healing sockets compared with controls. This was evidenced by thick trabeculae, highly vascularized bone marrow, and numerous osteocytes, whereas control sockets exhibited thin bony spicules and limited vascularity. 52

#### Clinical Efficacy of Black Cumin in the Treatment and Prevention of AO

Black cumin showed consistently positive clinical results in the included studies. Alabdullah et al reported superior healing outcomes compared with eugenol, 15 while Khan et al showed that the oil–powder mixture produced faster and more sustained pain relief than Alvogyl. 53 Al-Niaimi et al demonstrated comparable analgesic effects to Alvogyl by day 3. Additionally, the mixture of *Salvadora persica* (Miswak) and black cumin exhibited significant analgesic and anti-inflammatory properties in the treatment AO. 54 A previous study reported that black cumin nanoemulgel has been shown to enhance post-extraction wound healing by increasing fibroblast proliferation in the wound area. It may serve as a promising natural alternative for accelerating wound healing following tooth extraction. 55

These clinical outcomes reflect thymoquinone's combined antibacterial, anti-inflammatory, and pro-angiogenic effects, and suggest that black cumin may be one of the most promising herbal candidates for AO treatment among those reviewed.

#### Toxicity and Adverse Effects of Black Cumin

With topical use, an allergic reaction to black cumin ointment has been reported, manifesting as skin irritation, redness, itching, and, occasionally, the development of eczema or dermatitis upon contact. 56 Black cumin oil has been linked to severe allergic reactions, including acute contact dermatitis with lesions resembling erythema multiforme, Stevens-Johnson syndrome, or toxic epidermal necrolysis. Patients developed widespread polymorphic lesions, and histology revealed keratinocyte apoptosis with lymphocyte infiltration. Patch testing confirmed sensitivity, while thymoquinone and p-cymene were identified as potential allergens. 57

### Olive Oil

Olive oil ( *Olea europaea* L.), a member of the Oleaceae family, is classified by extraction methods that affect its polyphenol content, with extra virgin olive oil containing the highest levels of bioactive compounds such as oleuropein, oleocanthal, and hydroxytyrosol. 58 Its composition consists mainly of the saponifiable fraction (97–99%), dominated by oleic acid and other essential fatty acids, and a smaller unsaponifiable fraction rich in phenolics, triterpenes, and squalene. 59 These compounds provide antimicrobial, and anti-inflammatory effects, with oleuropein and hydroxytyrosol acting as potent free radical scavengers, and oleocanthal showing ibuprofen-like COX inhibition. 60

#### Antibacterial Properties of Olive Oil

Olive oil's antimicrobial components may help control bacterial colonization, lowering the risk of secondary infections at the extraction site, which is a major trigger for AO. One key antibacterial compound found in olive oil is oleuropein. Its antibacterial mode of action involves damaging the bacterial membrane and/or disrupting the cell's peptidoglycan structure. 61 Phenolic compounds in olive oil exhibit antimicrobial activity by disrupting bacterial membranes through proton release from hydroxyl groups. 62 Olive oil demonstrated bactericidal effects against *A. actinomycetemcomitans* and *F. nucleatum* and bacteriostatic effects against *P. gingivalis* . Its phenolic compounds also inhibited bacterial adhesion and biofilm formation at low concentrations by altering membrane permeability, impairing enzyme function, and causing protein leakage. 63

#### Anti-inflammatory and Analgesic Properties of Olive Oil

Oleocanthal exerts anti-inflammatory effects by inhibiting COX-1 and COX-2, thereby reducing prostaglandin synthesis in a manner similar to NSAIDs such as ibuprofen. It also downregulates proinflammatory cytokines, including IL-6 and TNF-α, supporting its potential role in controlling inflammation and promoting tissue repair in AO. 64

Oleocanthal inhibits COX-1 and COX-2 in a dose-dependent manner, showing greater potency than ibuprofen, while oleuropein aglycone suppresses TNF-α-induced MMP-9 expression in monocytes cell line. Hydroxytyrosol further reduces inflammation by downregulating IL-1β and TNF-α, without affecting IL-10 in animal model. 65

#### Wound-healing Properties of Olive Oil

The potential impact of various phenolic compounds found in olive oil on the wound-healing process has been demonstrated through findings supported by in vitro, in vivo, and some human studies. Olive oil phenolics promote wound healing by stimulating angiogenesis, fibroblast proliferation, collagen synthesis, and re-epithelialization. Experimental and clinical studies have shown improved healing in oral and cutaneous wounds, with enhanced tensile strength, neovascularization, and increased expression of TGF-β1 and VEGF-α. These effects highlight olive oil's potential to accelerate tissue regeneration and repair. 66 Topical olive oil extract significantly enhanced healing of traumatic oral ulcers in rabbits. Similarly, a clinical case report demonstrated its effectiveness in treating oral mucosal ulcers, showing reduced pain and inflammation with improved tissue regeneration. 67

#### Clinical Efficacy of Olive Oil in the Treatment and Prevention of AO

Only one included clinical study evaluated the use of olive oil for the management of AO. Khan et al compared intra-alveolar olive oil dressing with Nano-Bio Fusion (NBF) gel. 68 Both interventions resulted in progressive pain reduction and improved socket appearance over time; however, NBF gel demonstrated lower VAS pain scores at all recorded time points. By day 7, gingival healing and granulation tissue resolution were observed in both groups, although healing was graded as slightly more favorable in the NBF gel group. Despite this difference, olive oil still contributed to clinically meaningful pain relief and soft-tissue recovery, suggesting that its phenolic components and oleocanthal-related COX inhibition may support inflammation control and epithelial healing. Based on this single available study, olive oil appears to be a safe adjunctive dressing that can aid healing, though current evidence remains limited and less robust compared with other herbal products reviewed.

#### Toxicity and Adverse Effects of Olive Oil

In a mouse model, excessive intake of oxidized olive oil aggravated allergic contact hypersensitivity by promoting Th1 differentiation and increasing IFN-γ production. 69 Rare adverse effects have also been reported, including a case of olive fruit allergy, though the patient tolerated olive oil. 70 Overall, oral use of olive oil is considered safe for wound healing, with adverse events mainly limited to rare cases of allergic contact dermatitis from topical application. 71

## Medicinal Properties of Select Natural Compounds

All herbal compounds mentioned in the included studies showed the four pharmacological activities in common that contribute to the treatment of AO and the enhancement of extraction wound healing. These include anti-inflammatory and analgesic effects, wound-healing promotion, and antibacterial activity, as summarized in Table 3 .

**Table 3:** Summary of mechanisms of action from herbal remedies in treating alveolar osteitis (AO) and enhancing extraction wound healing

AO-related pharmacological properties	Mechanism of action from herbal remedies
Antibacterial	• Damage to cell walls and cytoplasmic membrane • Inhibit DNA and protein synthesis • Increase permeability of bacterial membrane which causes leakage of electrolytes • Produce intracellular ROS which inhibits bacterial growth • Inhibit quorum sensing • Inhibit the activity of membrane-bound ATPase which causes mitochondrial dysfunction • Inhibit biofilm formation • Reduce the expression of major virulence factor genes
Anti-inflammatory	• Inhibit expression of the iNOS and COX-2 via NF-kB and ERK/MAPK pathways • Inhibitors of proinflammatory mediators including IL-1β, IL-6, and TNF-α • Decreased leukocyte migration and adhesion • Increase the levels of the anti-inflammatory cytokine IL-10
Analgesic	• Inhibitory effect on VGSC and activation of TRPV1 • Suppress PGE _2_ production • Inhibit bradykinin production • Inhibit the activity of prostaglandins and leukotrienes • Reduce the levels of substance P in nerve endings
Wound healing	• Promote angiogenesis • Increase epithelial cell migration • Stimulate fibroblast activity to form collagen • Induce the expression of pro-angiogenic factors, including VEGF, TGF-β, FGF-2

Abbreviations: AO, alveolar osteitis; COX-2, cyclooxygenase-2 inhibitors; DNA, deoxyribonucleic acid; ERK, extracellular-signal-regulated kinase; FGF-2, fibroblast growth factor 2; IL, interleukin; iNOS, inducible nitric oxide synthase; MAPK, mitogen activated protein kinase; NF-kB, nuclear factor kappa B; PGE2, prostaglandin E 2; ROS, reactive oxygen species; TGF-β, transforming growth factor β; TNF, tumor necrosis factor; TRPV1, transient receptor potential cation channel subfamily V member 1; VEGF, vascular endothelial growth factor; VGSC, voltage-gated sodium channels.

The collective evidence from the included clinical studies indicates that herbal products possess therapeutic potential in the management of AO; however, the quality and consistency of evidence differ across agents. Black cumin and turmeric demonstrated the most robust and reproducible clinical benefits, with multiple studies reporting superior reductions in pain and inflammation and more rapid soft-tissue healing when compared with conventional dressings containing eugenol or Alvogyl. *Aloe vera* also showed favorable clinical performance, particularly in enhancing epithelial regeneration and providing effective analgesia, supporting its role as a well-tolerated adjunct to standard care. In contrast, evidence for olive oil remains limited to a single clinical trial, in which it produced satisfactory healing outcomes but was comparatively less effective than a commercially available bioactive gel, underscoring the need for further controlled studies to clarify its clinical relevance. Clove, while historically foundational in AO therapy, primarily served as a reference comparator in the reviewed studies and did not demonstrate superiority over the other evaluated herbal interventions.

## Conclusion

Alveolar osteitis remains a frequent postoperative complication, and effective management requires both pain control and support of socket healing. The evidence reviewed indicates that black cumin, turmeric, and *Aloe vera* provide clinically meaningful benefits through combined anti-inflammatory, antimicrobial, and wound-healing effects, often performing as well as or better than conventional eugenol-based dressings. Olive oil demonstrated favorable healing in the single study available, but evidence remains limited, and further trials are needed to confirm its clinical value. Clove continues to serve as a traditional reference treatment but did not show superior outcomes compared with other herbal agents. Overall, herbal products show promise as adjunctive or alternative options for the management of AO; however, standardized formulations and high-quality randomized controlled trials are required to support routine clinical implementation.
